# Testing Digital Methods of Patient-Reported Outcomes Data Collection: Prospective Cluster Randomized Trial to Test SMS Text Messaging and Mobile Surveys

**DOI:** 10.2196/31894

**Published:** 2022-03-17

**Authors:** Anish K Agarwal, Zarina S Ali, Frances Shofer, Ruiying Xiong, Jessica Hemmons, Evan Spencer, Dina Abdel-Rahman, Brian Sennett, Mucio K Delgado

**Affiliations:** 1 Department of Emergency Medicine University of Pennsylvania Philadelphia, PA United States; 2 Leonard Davis Institute University of Pennsylvania Philadelphia, PA United States; 3 Penn Medicine Center for Health Care Innovation University of Pennsylvania Philadelphia, PA United States; 4 Department of Neurosurgery University of Pennsylvania Philadelphia, PA United States; 5 Behavioral Science and Analytics for Injury Reduction University of Pennsylvania Philadelphia, PA United States; 6 Department of Orthopedic Surgery University of Pennsylvania Philadelphia, PA United States

**Keywords:** patient-reported outcomes, mobile surveys, research methods, text messaging, mobile survey, data collection, patient engagement, response rate

## Abstract

**Background:**

Health care delivery continues to evolve, with an effort being made to create patient-centered care models using patient-reported outcomes (PROs) data. Collecting PROs has remained challenging and an expanding landscape of digital health offers a variety of methods to engage patients.

**Objective:**

The aim of this study is to prospectively investigate two common methods of remote PRO data collection. The study sought to compare response and engagement rates for bidirectional SMS text messaging and mobile surveys following orthopedic surgery.

**Methods:**

The study was a prospective, block randomized trial of adults undergoing elective orthopedic procedures over 6 weeks. The primary objective was to determine if the method of digital patient engagement would impact response and completion rates. The primary outcome was response rate and total completion of PRO questionnaires.

**Results:**

A total of 127 participants were block randomized into receiving a mobile survey (n=63) delivered as a hyperlink or responding to the same questions through an automated bidirectional SMS text messaging system (n=64). Gender, age, number of comorbidities, and opioid prescriptions were similar across messaging arms. Patients receiving the mobile survey were more likely to have had a knee-related surgery (n=50, 83.3% vs n=40, 62.5%; *P*=.02) but less likely to have had an invasive procedure (n=26, 41.3% vs n=39, 60.9%; *P*=.03). Overall engagement over the immediate postoperative period was similar. Prolonged engagement for patients taking opioids past postoperative day 4 was higher in the mobile survey arm at day 7 (18/19, 94.7% vs 9/16, 56.3%). Patients with more invasive procedures showed a trend toward being responsive at day 4 as compared to not responding (n=41, 59.4% vs n=24, 41.4%; *P*=.05).

**Conclusions:**

As mobile patient engagement becomes more common in health care, testing the various options to engage patients to gather data is crucial to inform future care and research. We found that bidirectional SMS text messaging and mobile surveys were comparable in response and engagement rates; however, mobile surveys may trend toward higher response rates over longer periods of time.

**Trial Registration:**

ClinicalTrials.gov NCT03532256; https://clinicaltrials.gov/ct2/show/NCT03532256

## Introduction

Health care delivery continues to evolve, with an effort being made to create patient-centered care models. Learning health systems engage patients and elicit patient-reported outcomes (PROs) data to continuously improve and drive clinical practice [1,2]. Incorporating PROs into clinical decision-making has led to improvements in patients’ quality of life, improved communication, and reductions in unscheduled care [3-5]. Collecting PROs has remained challenging and their integration into clinical decisions remains understudied [6].

The use of mobile technology (cell phones, smartphones, tablets) continues to increase in society and health [7,8]. Engaging patients to capture PROs may provide clinicians with additional understanding to support care and motivate behavior remotely [9,10]. As digital strategies continue to expand, there has been limited research on the optimal ways to reach patients and capture PROs. Research using mobile health has grown in the past decade [11-13]. Clinicians have attempted to use mobile technology to track medication adherence [14,15], encourage healthy behaviors [16], improve home monitoring [17], and institute automated “hovering” to track chronic diseases [18]. However, less is known about the various mobile methods of engaging with patients and collecting and monitoring patient-reported data.

Within the context of the opioid epidemic and in an effort to promote opioid prescribing stewardship, surgical studies have collected PROs focused on pain intensity and opioid prescribing and use through surveys to reduce excessive prescribing [19-22]. These studies have revealed a mismatch between prescribing and patient-reported use, but are limited by their retrospective design, recall bias, and limited response rates. PROs have been used in orthopedic surgery to help guide preoperative decision-making and improve patient satisfaction [9,23]. To overcome the limitations of prior research, the existing gap regarding prospective PRO data on pain, function, and opioid consumption must be addressed. The rapid evolution of mobile technology may provide an opportunity for clinicians to assess trends in patients’ self-reported pain and use of prescription analgesics, test methods to support safe and effective pain management, and translate data into clinical protocols [24].

The purpose of this study was to prospectively investigate two mobile patient engagement strategies to collect PRO data. Surgeons seek PRO data on acute pain management to inform safe opioid prescribing and to effectively manage acute pain. We compared response and completion rates of postoperative PROs among patients using bidirectional SMS text messaging versus mobile hyperlink surveys. To our knowledge, this is the first study to compare two distinct mobile engagement approaches in this context. The hypothesis of this study was that conversational SMS text messaging would result in higher response and completion rates as compared to mobile survey hyperlinks.

## Methods

### Overview

This was a prospective, block randomized trial of adult patients (aged 18 years or older) undergoing elective orthopedic procedures (ClinicalTrials.gov NCT03532256). The study took place over 6 weeks between July 1, 2019, and August 12, 2019. The primary objective was to determine if the method of patient engagement would impact response and completion rates. The primary outcome was response rate and total completion of PRO questionnaires.

Eligible patients included adult patients undergoing an elective outpatient orthopedic surgery (including knee, hip, shoulder, and elbow) and prescribed an acute opioid for postoperative pain. Acute opioid pain medications included oxycodone, oxycodone/acetaminophen, hydromorphone, or hydrocodone. Exclusion criteria included no access to an SMS-capable device or no opioid prescription. These inclusion and exclusion criteria were consistent with prior published research protocols using an established automated postoperative messaging program [24,25]. All patients were recruited from the University of Pennsylvania Health System Department of Orthopedics, which performs approximately 13,000 surgeries per year. In addition to general procedures (~3500 annual surgeries), the department consists of the following divisions: (1) Sports Medicine (~1700 annual surgeries), (2) Hand (~1900), (3) Joints (~4500), and (4) Trauma (~1500).

The research team had previously worked with the institution’s legal, privacy, and patient safety departments to obtain remote, SMS text message–based consent for data collection [25]. This was an intentional design of a larger institutional program aimed at improving acute opioid prescribing. The approach allows for remote consent in an attempt to reduce clinical providers’ workload and improve the scale of engaging eligible patients.

Following an elective outpatient surgery within the Department of Orthopedics, patients who underwent surgery received an initial SMS text message on the second day after the procedure. This message informed the patient of safe SMS text messaging data practices, provided links to further information regarding the follow-up research study, and offered the ability to opt in or opt out of further messaging. Patient were asked to consent via a simple SMS text message response of “yes.” Consenting patients were then sent SMS text messages on postoperative days number 4, 7, 14, 21, and 28. Patients were block randomized in groups of 2 or 4 to receive either (1) a hyperlink to a web-based mobile survey or (2) automated bidirectional SMS text messaging in a conversational format. Mobile survey questions and conversational questions were identical and included PROs related to self-reported pain intensity, ability to manage pain, use of prescribed medications, and ability to control pain. Block randomization was used to achieve balance in the two groups across demographics, procedure date, and dates of mobile engagement.

Individuals who either did not respond, opted out, or self-reported no further planned use of acute opioid medications were not subsequently messaged. For example, if a participant completed the mobile survey or replied to the bidirectional SMS text messaging on postoperative day 4 and indicated they were no longer planning on using their opioid prescription, no further messaging was sent on day 7, 14, 21, or 28. Participants who did not respond to any messaging would receive a reminder message within 30 minutes; if participants did not respond, then no further messaging was sent. The follow-up intervals were determined with input from a key clinician and surgeon and were in line with prior studies that evaluated PROs at 1 or 2 weeks postoperation.

Descriptive summary statistics were used to characterize the study population, using mean and standard deviation for age, and frequencies and percentages for categorical variables including gender, type of surgery, opioid tablet quantity, comorbidities, and invasiveness of the procedure (open surgical approach vs laparoscopic). To determine differences in the primary outcome of response rate at day 4 between the messaging arms and categorical demographic variables, Fisher exact test was used. To determine the difference in response at day 4 by age, Student *t* test was used. To determine if there were differences in secondary outcomes such as opioid use over time, Kaplan-Meier product-limit method with a log-rank test was used. A 2-sided α of .025 was considered statistically significant. All opioid prescription types were converted to equivalent doses of 5 mg oxycodone tablets [26]. All analyses were performed using SAS statistical software (version 9.4; SAS Institute).

### Ethics Approval

The Institutional Review Board of the University of Pennsylvania approved this study (number 827461).

## Results

Over 6 weeks, 127 participants were block randomized into either receiving a mobile survey (n=63) delivered as a hyperlink or responding to the same questions through an automated system—a chatbot using bidirectional SMS text messaging (n=64). Overall, 57.4% (n=73) of participants were male, the mean age was 38.5 (SD 13.9) years, and 70.8% (n=90) had had a knee procedure. Gender, age, number of comorbidities, and number of opioid tablets prescribed were similar across arms (Table 1). Patients receiving the mobile survey were more likely to have had a knee procedure (n=50, 83.3% vs n=40, 62.5%; *P*=.02) but less likely to have had an invasive or open procedure (n=26, 41.3% vs n=39, 60.9%; *P*=.03).

Overall engagement over the immediate postoperative period was similar between the messaging arms (Table 2). Using automated bidirectional SMS text messaging, the overall response rate was 45.3% (29/64) versus 49.2% (31/63) using a hyperlink to a mobile survey. For those patients using opioids in the past 4 days, prolonged future engagement was higher in the mobile survey arm at day 7 (18/19, 94.7% vs 9/15, 60%). Among nonresponders, the majority of patient drop-off occurred at day 4. Patients with more invasive or hip procedures showed a trend toward being responsive at day 4 as compared to those not responding (Table 3).

**Table 1:** Patient demographics.

Demographic		Bidirectional SMS text messaging (N=64)	Mobile survey (N=63)	*P* value	
**Sex, n (%)**					.72
	Female	26 (40.6)	28 (44.4)		
	Male	38 (59.4)	35 (55.6)		
Age (years), mean (SD)		37.8 (14.2)	39.2 (13.7)	.58	
**Comorbidities, n (%)**					.49
	0	29 (45.3)	22 (34.9)		
	1-2	13 (20.3)	16 (25.4)		
	>2	22 (34.4)	25 (39.7)		
**Type of surgery, n (%)**					.02
	Knee	40 (62.5)	50 (83.3)		
	Shoulder	19 (29.7)	6 (10)		
	Hip	5 (7.8)	4 (6.7)		
	Elbow	0 (0)	2 (3.2)		
Invasive procedure (open or nonlaparoscopic), n (%)		39 (60.9)	26 (41.3)	.03	
**Quantity of opioid tablets^a^ prescribed, n (%)**					.09
	<11	15 (23.4)	23 (36.5)		
	11-20	25 (39.1)	28 (44.4)		
	21-30	19 (29.7)	8 (12.7)		
	>30	5 (7.8)	4 (6.4)		

^a^Opioid tablet in 5 mg oxycodone equivalents.

**Table 2:** Patient-reported outcomes and response rates.

Day	Conversational messaging						Mobile survey				
	Total, n	No response, n	Completed^a^, n	Continue on^b^, n	Response rate, %	Total, n		No response, n	Completed^a^, n	Continue on^b^, n	Response rate, %
Day 4	64	28	21	15	56.3	63		30	14	19	52.4
Day 7	16	7	5	4	56.3	19		1	10	8	94.7
Day 14	4	0	3	1	100	8		1	5	2	87.5
Day 21	1	1	0	0	0	2		0	1	1	100
Day 28	0	N/A^c^	N/A	N/A	N/A	1		0	1	0	100
Final response	N/A	34	30	N/A	46.9	N/A		32	31	N/A	49.2

^a^No longer taking opioids or no longer planning to take them.

^b^Participants indicating continued or planned use of opioids, who were thus sent additional surveys on subsequent days.

^c^N/A: not applicable.

**Table 3:** Response at day 4 by demographics.

Demographic			No response (N=58)		Responded day 4 (N=69)		*P* value	
**Arm, n (%)**								.72
	Bidirectional messaging	28 (48.3)		36 (52.2)				
	Mobile survey	30 (51.7)		33 (47.8)				
**Sex, n (%)**								.37
	Female	22 (37.9)		32 (46.4)				
	Male	36 (61.1)		37 (53.6)				
Age (years), mean (SD)			38.1 (13.8)		38.9 (14)		.76	
**Comorbidities, n (%)**								.74
	0	23 (39.7)		28 (40.6)				
	1-2	15 (25.9)		14 (20.3)				
	>2	20 (34.5)		27 (39.1)				
**Type of surgery, n (%)**								.09
	Knee	44 (75.9)		46 (66.7)				
	Shoulder	12 (20.7)		13 (18.8)				
	Hip	1 (1.7)		9 (13)				
	Elbow	1 (1.7)		1 (1.5)				
Invasive procedure (open or nonlaparoscopic), n (%)			24 (41.4)		41 (59.4)		.05	
**Quantity of opioid tablets^a^ prescribed, n (%)**								.69
	<11	20 (34.5)		18 (26.1)				
	11-20	24 (41.4)		29 (42)				
	21-30	11 (19)		16 (23.2)				
	>30	3 (5.2)		6 (8.7)				

^a^Opioid tablet in 5 mg oxycodone equivalents.

## Discussion

### Principal Findings

This study has two key findings within the context of mobile patient engagement and data capture. First, immediate postoperative patient engagement and research consent are feasible using digital methods. Second, there were no significant differences in the overall response rates between the modalities of bidirectional SMS text messaging and mobile surveys. We deployed a direct-to-patient approach in obtaining mobile consent to prospectively capture PROs for postoperative pain and pain management. Prior studies aiming to understand patients’ pain and use of prescription opioids following surgeries have been limited by retrospective design, telephone or paper surveys, and recall bias [19,22,27]. We worked collaboratively within the health system to develop an approach that offloads clinical providers from obtaining written consent during preoperative visits and reaches out to patients following their procedure through SMS text messaging. This mobile consent process may be further studied to decrease in-person time, allow for researchers to link important research and other patient information, and offer patients the ability to opt out.

Second, the method and approach used to capture patient-reported data may not significantly impact initial response rates and overall completion rates. The ways in which patients use digital technology and mobile apps continue to change [28]. This study begins to analyze direct-to-patient methods that capture patient-reported data [29]. These early results indicate mobile methods can be used to engage postoperative patients and may provide a scalable approach for engaging larger patient populations. Traditional paper-based surveys rely on mail services and may introduce time delays and recall bias, whereas digital methods allow patients to respond in the moment [30]. Though not statistically significant, we found that patients undergoing more invasive procedures (ie, open surgeries and nonlaparoscopic surgeries) or any procedure on the hip were more likely to respond and remain engaged. This suggests an opportunity to provide tailored content and messaging as more procedures become outpatient and more recovery is based in the home. Ultimately, we describe similar and consistent completion rates as the weeks passed following patients’ surgeries.

### Limitations

This study has limitations. First, the study and data collection were performed at a single academic medical center and thus the study is not generalizable. Second, selection bias may be present as patients needed to have a mobile device and opt to answer questions through either the mobile survey or the bidirectional SMS text messaging system. Those patients receiving a mobile link to the survey must have a smartphone and may represent a select population. Third, patients were undergoing elective outpatient procedures and may represent a population that is not generalizable to all procedures. This study compares two techniques that have been rapidly adopted; it does not compare these directly to paper methods, which have more traditionally been used.

### Conclusion

This study demonstrates the early feasibility of PRO capture using two methods of patient engagement following orthopedic surgery. The findings suggest no major differences between bidirectional SMS text messaging and mobile surveys to collect PRO data. In an increasingly digital era, clinicians and researchers may employ digital surveys as a tool for rapid collection of PROs, which can inform research and a learning health system. Future studies will need to investigate larger scale programs and generalizability outside of surgical settings and for broader populations.

## Appendix

Multimedia Appendix 1 CONSORT eHEALTH Checklist (V 1.6.1).

## Abbreviations

PRO: patient-reported outcome
